# American Dental Association

**Published:** 1891-12

**Authors:** 


### Reports of Society Meetings.

---

### AMERICAN DENTAL ASSOCIATION.

*August 5, 1891.—Second Day.—Morning Session.*

Meeting called to order at 9.40 o'clock.

The President announced that the paper read by Dr. Custer, of Dayton, Ohio, was open for discussion. There being none, the following paper was read by Professor Peirce:

### THE DENTITION OF THE FELIDÆ.

BY ALTON H. THOMPSON, D.D.S., TOPEKA, KANSAS.

The dentition of the true cat presents an interesting subject for study, on account of being a conspicuous example of a highly specialized dental organization. The extremes of form among the teeth of animals indicate the great possibilities of variation, and thereby throw light on less highly specialized forms, in which extremes are modified, and compromised forms or composites are developed which are adapted to more or less mixed diets.

Special and extreme forms of diet, of course, lead to the evolution of special and extreme dental organizations, which must necessarily be adapted to the food employed, and which the animal prefers to employ.

The carnivorous animals, having become adapted through countless generations to an exclusive dietary,—the flesh of other animals,—have in their dental organizations been modified and specialized to accommodate them to such a diet, and present forms peculiarly and specially adapted to it. In other highly specialized forms, as the herbivora, of course, the same rule holds true, but it does not hold with animals of a mixed diet, in which the dentition is modified and extremes are reduced and compromised to adapt the teeth to varied foods.

In the true Felidæ, as in the true herbivora, however, the teeth are extremely modified to adapt them to an extreme diet, and this class, therefore, presents an interesting example of high specialization for study.

The whole order of the carnivora, or flesh-eaters, embracing cats, dogs, bears, and their allies, present a high order of development.

"The canine on each side is very much lengthened, is sharply pointed, and recurved for seizing, holding, and tearing prey. The upper canine is separated from the incisors by an interval,—a diastema,—the lower canine being received into this space. The incisors are small, short, blunt, and always six in number, and stand nearly in a straight line across the front of the jaw. The outer incisor is sometimes large and pointed like the canine. These anterior teeth are tolerably uniform throughout the order, but the molars vary much in form and number, in accordance with the diet.

"In the most purely carnivorous members of the order, the Felidæ, the true molars are reduced in number on each side, and the cusps are produced into long cutting blades,—the sectorial teeth. In the bears, on the other hand, the molars are more numerous, and are furnished with obtuse and rounded cusps. The fourth premolar in the cat is the largest of the molar series, and is the main cutting tooth. The crown is divided into two parts,—the one a thin, sharp-edged blade which runs in an antero-posterior direction and is sometimes divided by notches into two or more cusps; the other part, the tubercle, is a shorter and blunt cusp, situated on the inner side of the anterior end of the blade. In the pure flesh-eaters the blade is well developed, and the tubercle of less size than in the mixed feeders. The lower tooth opposed to this is the first true molar, and in the Felidæ consists solely of a blade, which is divided into two large cusps and a rudimentary cusp behind."[1]

In a general way, the characters which indicate a pure flesh diet are undersize of the incisors, large, deeply-implanted canines, wide diastema, reduction of the molar series, cutting blades on the molars, etc.

The true molar is a small tooth, and is scarcely visible from the outside of the jaw. The lower first molar consists of a blade only divided by a V-shaped notch. The first premolars are much reduced and often are scarcely visible. The formula of the family of the Felidæ is as follows:

$$\text{I. } \tfrac{3}{3} = \text{c. } \tfrac{1}{1} = \text{p. m. } \tfrac{3}{2} = \text{m. } \tfrac{1}{1} = 15 = 30.$$

"The blades of the molars pass each other like the blades of scissors in express relation to cutting of flesh. The compressed trenchant part of the molars forms a sharp edge, more or less deeply cleft in different genera. This blade is usually accompanied by one or more basal tubercles. The blade of the upper sectorial tooth always

[1] Charles Tomes's "Dental Anatomy," p. 375.

lays upon the outside, and a little in advance of the lower sectorial. The upper permanent sectorial succeeds and displaces a deciduous tubercular molar in all carnivora, and is therefore essentially a premolar tooth. The lower sectorial comes up behind a deciduous series, and has no immediate predecessor, and is therefore a true molar. In no other groups of animals are the jaws so well and so variously armed with dental organs. The vacancies are only sufficient to allow of the interlocking of the strong canines, which are formidable and efficient weapons for seizing living prey, and lacerating and dividing flesh. The incisors are well adapted for scraping and gnawing bone, the sectorials for cutting flesh and dividing resisting fibrous tissues, and the tubercles for cracking and crushing bone. The jaws are short, the muscles strong, the articulation a mere hinge, so that it limits jaw movements to the vertical alone."[1]

There is not much mastication or insalivation of food in this order, as the flesh food is readily digested. The simple stomach and short intestine indicate also that they subsist on a food easily digested. The whole alimentary system indicates a simple diet which requires but little preparation for assimilation. It is simplicity itself, when compared with the complicated masticating apparatus and extensive digestive system of herbivorous animals, whose diet is resisting and unfit for assimilation, and requires extensive manipulation to prepare it for the building of animal tissue.

It is highly interesting to pursue the subject into the field of evolution, and to study the process by which such a highly specialized organization arose.

Professor E. D. Cope ("Origin of the Fittest," page 363) says, "The primitive dentition has been modified in two directions,—viz., to form teeth for grinding and a sectorial dentition. The specially developed teeth of the carnivora are the canines and sectorials. The successive modifications of forms which have resulted in the existing specialized lower sectorial tooth of the Felidæ consists in the gradual obliteration of the internal and posterior tubercles, and the enlargement of the external anterior tubercles in connection with an additional anterior tubercle.

"The modification of the characters of the dentition, taken as a whole, consists in the reduction of the number of the teeth and the manner of their specialization.

---

[1] Richard Owen, "Odontography," 1840, p. 490.

"Observation of the movement of the jaws of the carnivora shows that they produce a shearing motion of the inferior against the superior teeth. This is quite distinct from the sub-horizontal movements of ruminants, or the vertical motion of hogs or monkeys. In the crowns of the sectorials, the inner side of the superior and the outer side of the inferior, are worn in the process of mastication.

"The cutting of cartilages and tough tissues is best accomplished by the shearing of the outer edge of the lower molars on the inner edge of the external tubercles of the superior teeth in simple tuberculate teeth, the jaw being too wide to shear on the inner side of the tubercle of the superior series, so the cusps of both upper and lower teeth which engage in this process are developed in elevation at the expense of those not engaged in it,—*i.e.*, the internal cusps of the same teeth.

"The atrophy of the latter are not due to friction, but to disuse, while the effect of use has lengthened the external cusps in the carnivora, and has resulted in a plain, grinding surface in the ungulata. The position of the sectorial tooth is just at the front of the masseter muscle, where the greatest amount of force can be brought to bear upon it. The last molars are not modified in the sectorial teeth in the modern carnivora, as they are in some extinct forms in the development of the prehensile character of the canine teeth.

"The prehensile character of the sectorial teeth or the premolars was lost. Extinct forms snapped their prey, as is seen in some existing dogs with long jaws, which do not permit of the lacerating power of the canines, as in the Felidæ. The latter have the masseter muscle situated more anteriorly, in order to render the canines more effective. This muscle has advanced from behind forward in the development of the carnivorous types."

Dr. John A. Ryder, in describing the mechanical genesis of tooth-forms, says,—[1]

"The dentition of the carnivora or feræ is bunodont, the jaws wide, with the tubercles latterly compressed into sharp edges to cut prey. The mandibular movement is ginglynoid, and admits of the least lateral movement; all that is admissible is that which is effected by the lateral sliding of the cylindroid condyles in the glenoid cavities, but the effect is, however, widely different from the lateral movement observed in the ungulata.

---

[1] Proceedings of the Academy of Natural Sciences of Philadelphia, 1878, p. 48.

"The passage from the archetype bunodont tooth to the scissor-like (carnassial) sectorial form is plainly exhibited by selecting a series leading from the lowest of both ancient and modern forms up to the Felidæ as the centre of specialization. The characteristic cylindroid condyle of the cat is probably formed to resist the pulling action which that animal exhibits when holding down prey with the paws, as this prevents the drawing of the jaw forward."

This compels the single movement of opening and closing the jaw, and this gives rise to the peculiar form of tooth with sharp, blade-like cusps so well adapted to the food employed. The tooth is developed in a vertical direction on account of vertical movement, but latterly, as in the herbivora, which have lateral movement of the jaws. Types of teeth have been developed in accordance with the amount and direction of mandibular movement, and the teeth of the Felidæ are formed in accordance with this rule. Cusp duplication is effected by this movement, as pointed out by Professor Cope and Professor Harrison Allen (*Dental Cosmos*, vol. xvi. p. 617), by the thickening of the tooth in a lateral longitudinal or oblique direction in obedience to the growth of force.

"The development of a bicuspid or quadricuspid tooth is effected by repetition of the cuspid or incisor forms by the functional development of the cingules at the base of the crowns, which are developed in the cusps."

In this manner repetition of cusps arose and duplication became possible. In the ruminating ungulates this duplication is more extensive than in the carnivora, in which a simplicity in this respect is maintained, but a growth of the cusps is manifested for a special purpose.

The most highly specialized teeth in the Felidæ are therefore the sectorial premolars, and the prehensile canines, which are characteristic. Those teeth place this family at the head of the carnivora as the most highly specialized members of the order. They furnish an example of dental variation that is quite remarkable when studied in its minutiæ. Other orders furnish examples of extreme specialization, which are also quite interesting and instructive.

*The President.*—Gentlemen, Dr. Corydon-Palmer wishes to say a few words to you.

Dr. Corydon-Palmer then unveiled a portrait of the late Dr. William H. Atkinson.

*Dr. Corydon-Palmer.*—I think the portrait will speak for itself. It was painted by Mr. J. W. Bell, who is a particular friend of mine. It shows Dr. Atkinson as he was in life, when we knew him and loved him most. I present it here with the hope that some time we may have a place where such valuable things can be taken care of, and, if there ever should be, this may be had for the beginning of a collection of portraits. There are portraits of the oldest practitioners in this country, and I would propose that copies of them be made, and that they should be arranged at some place so that we may have them to look upon; that we may remember them as we have seen them in life.

*The President.*—Gentlemen, Dr. Palmer spoke to me last night, —that he had a matter to present to this society. I told him that in consideration of his being one of the old members of this association that I would grant him that privilege. I am sure you are all gratified to see the likeness of our first-elected president. This is, I understand, a gift to this society, when we have a place where we can hang it, and I think that Dr. Palmer should receive our thanks for this pleasant surprise.

Dr. Abbott moved a vote of thanks.

Carried.

DISCUSSION.

*Dr. Barrett.*—The presentation of such a paper as this and its passage without any discussion would seem to show indifference on the part of intelligent dentists. It seems to me that if we profess to be scientific men we must consider something besides that from which we earn our daily bread. Of course, we are studying human teeth, because we are always at them. That is professional work, not scientific; but if we consider the dentitions generally, of the whole order of vertebrates, then we are doing scientific work, and this paper is a scientific one, strictly and technically. It is not a practical paper; it is not professional work. It is in the line of true science, and therefore I desire the chance to say something upon it.

Dr. Thompson has followed Huxley mainly. Most homologists have followed the classification of Cuvier as the proper one, still in the latter days his system of division of the teeth and his method of regarding them has fallen into decadence.

The sectorial teeth, of which the paper treats so much, are between the last molar and the first premolar, which have become specialized in certain orders; but Cuvier's system of regarding the teeth was somewhat different from this. We divide the teeth into incisors, cuspids, premolars, and molars, molars being those which succeed the deciduous molars; but Cuvier's formula divided them into incisors, cuspids, false molars, carneous, and tubercular molars.

The carneous are those which are called sectorial teeth. In Cuvier's system there are no sectorial teeth, consequently he is obliged to introduce another kind of teeth, which he calls false molars, and it seems to me that therefore his system is somewhat weak.

If there be a great archetype of dentition, it would be, in my opinion, that of forty-eight teeth. I do not say that all the dentitions have been modified from some great archetype. The true archetype to-day of the ungulata is probably forty-four, but I believe that the great archetype is forty-eight, and in that case the formula would be written in this way:

$$\frac{3-3}{3-3}\ \text{c.}\ \frac{1-1}{1-1}\ \text{p. m.}\ \frac{4-4}{4-4}\ \text{m.}\ \frac{4-4}{4-4} = 48.$$

There are six incisors; the premolars are 4—4, the molars are 4—4, equalling forty-eight teeth.

It is not at all certain but that the incisors are 4—4, but if there be a typical dentition that is it.

In the Felidæ, in which the teeth have become so materially changed, there has been a dropping from this end of the mouth; from the anterior portion of the mouth there have been none lost. Then we have the premolars modified, and we have the molars drop to 1—1, and the sectorial teeth are the last of these, the third molar and the first true molar.

In the Owen method he has changed that, and has made the last premolar; that tooth does not succeed a deciduous molar, consequently I do not believe it to be a premolar. How is it that from the posterior portion of the mouth so many have been lost, while on the anterior side there have been none lost? The premolars being small, and there being space between them, how comes it?

Most of the homologists claim, and I think Dr. Thompson does, that it is through the selection of the food that the sectorials are produced,—by the food upon which they live; but the question comes back to us, Which was first, the egg or the hen? and it is a question that I do not know can be perfectly answered to-day.

Did the first fowl come from the egg or did the egg come from the fowl? It must necessarily follow that one of them was first, but which was it? If the doctrine of evolution be true, there may have been an evolution from some other form.

In the canines, which are related to this class, we have a still less modified dentition than in the Felidæ; and the dentition of the canines equals forty-two. In the canines we find that the modification is less than in the Felidæ, but there is no reason to suppose that the canines preceded the Felidæ, and that the Felidæ are a still further modification from the great archetype of the dentition than are the canines.

Cuvier divided the feline dentition into the incisors, the canines, the false molars, the carneous, and the true molars; and he has in the molar system the false molars, 3—3, 4—4; the carneous, 1—1, 1—1; the tubercular molars, 2—2, 2—2; consequently he has introduced the carneous molar there in place of the sectorial tooth.

In this dentition, taking that as the great archetype, we see that in the human dentition there has been lost a molar on each side,—you have lost two premolars and one incisor from each side. That some of the teeth are, and that the wisdom tooth is now, in a condition of being entirely suppressed, seems to me quite probable. You may say that during the historical period there have been no indications of its suppression, but the historical period is but a drop in the ocean of time that has fled during the past. It is almost as nothing, and the time is altogether too short to trace any peculiar modifications. I have seen repeatedly in the gorilla a fourth molar that was not at all a molar, that could be considered in any sense a supernumerary molar. It is sometimes found in the human family. It is not in any sense a supernumerary tooth, but evidently belongs in the perfect succession, for it has all the perfection of any of the molars, so that it would look as though there had been a suppression here; and that there has been a suppression in incisors is proven by the occasional appearance of a third incisor, so that there is undoubtedly a modification in the human dentition.

There may also have been a modification of dentition in the Felidæ. Understand, gentlemen, I am not here to urge the claims of evolution by any means; and there are very few that understand evolution exactly as I believe it to be, and as most of the authorities do. For instance, some gentlemen suppose that evolution means, "Was your grandfather a monkey?" It has nothing whatever to do with that. The true doctrine of evolution would be this: that from a single organic cell there began a differentiation and development which resulted finally in the Felidæ. In another direction it resulted in the ungulata; in another, in the rodentia; in another direction it resulted in man; but that man was ever a monkey or a long-tailed rat is not proven at all.

But that the dentition has been modified by surroundings is clearly and unmistakably proven, and if the dentition has been modified by the environments, it is quite probable that the form and shape of the teeth may have been changed also.

Owen's method is from the forms or shapes of the teeth; that is not entirely perfect. The shape of the tooth does not always indicate its offices.

I do not know of any class of men that are more competent to follow this line of study, provided they pay attention to it and are sufficiently educated for it, than the dentists. None have a better opportunity to study it; to none is it so near in their daily work as to the dentists; and if the problem is to be solved, I think the dentists should solve it, and the direction of the professional mind should tend to the clearing up of this scientific question. We have been altogether too practical. We should get to a point where we can look at something besides amalgam and red rubber. We should consider some higher problems and put ourselves upon the plane of science, and then we shall be accepted by scientific men, but until then we cannot consider ourselves as studying science in any way.

*Dr. Peirce.*—When a paper such as Dr. Thompson's is read before a body of specialists, the query comes at once, Can we draw a lesson from it that has application to our daily labors? Can we make it available in our specialty? In the few moments that I have upon this floor, I want to see if that is possible. We have learned from our previous studies, as well as from the paper just read, that the carnivora embraces three or four varieties. They are all either flesh-eaters or fish-eaters. They vary in their habits and in their environments, and in proportion as they vary in their food habit and means of getting their diet, so do their teeth and the shape of their heads vary, although they have a general resemblance, being all classed with the carnivora. We have this order—the carnivora—divided into the terrestrial, the aquatic, the arboreal, and the fossorial. And though feeders upon flesh and fish, they vary, and just in proportion as they have become specialized in their life habits, their teeth have become thoroughly adapted and fixed in shape and number with a recognized tendency to a reduction in the number. This is apparently the law; as an animal becomes specialized in his habits, the number of teeth are never increased, but invariably reduced, as compared with those of the ancestral type.

This fact I want noted as recognized in several mammalian forms; you all know that the typical number of teeth for the horse has been forty-four, and it is also stated as the typical number for the mammalia. The modern, and especially the thorough-bred, horse has four of these teeth becoming rudimentary; so he has but forty permanent teeth. The specialization of that animal has cost it four teeth. Whether that is due entirely to diet and non-use, or to the intensification of the nervous system and diversion of nutrition, has never been settled. I think probably it is as much due to the one as to the other.

You are also familiar with the fact that man belongs to the order of mammalia, and indulges in an omnivorous diet. By virtue of that diet the teeth are asking, Which way? Are we settling down to a specialization that can better adapt these organs to their needs, to their function? My own impression is that one of the principal factors in the decay of the human teeth is because they are in a transitional condition. The human family is not yet old enough or enough limited in diet to have them become specialized, and so our teeth are waiting and, as I just said, asking, "Which way?" and until we can say to them, "This is our diet, and shall be our diet for hundreds or thousands of years," we will have that uncertainty in their durability and number.

Towards a reduction in number you all know that the human family is looking by the loss of the four third molars. Thirty-two is the number we count. How many patients have we with but twenty-eight? Thousands of them, I imagine. Then, again, let us take the races that live entirely upon vegetable food,—rice, as the Chinese; with them we find a very marked suppression in the cuspid or canine teeth. I assert this is true because of the absence of a meat diet. These are carnivorous teeth. We find with this limited and exclusive diet a marked increase in size of the incisors, to compensate for the marked characteristic suppression of the canines. I make this remark based upon the observations of others and upon conversation with many who have travelled and worked among these people. It therefore must be taken with the degree of uncertainty belonging to such sources of information.

If it be true, however, it is in this same evolutionary line; but instead of dentists suggesting, as often has been the case, that the human family will eventually become edentulous, let us be assured that the human family will have just the number and shape of teeth needed for its habits. If you can pick out from patients families who live upon a solid diet,—upon dry foods,—and use their teeth for mastication, and saliva for insalivation without washing it into the stomach, minus these essential requirements, you will select the families with whom you have the least trouble in saving their teeth, and in whose teeth fillings have greater durability than others, and whose mouths will be purer and cleaner in every respect than those who pursue the opposite course.

*Dr. Carroll.*—I did not have the pleasure of hearing the paper read. I very much regret it, but I have had the pleasure of listening to the remarks of the gentleman from Buffalo. I wish to make one observation touching the remark of Dr. Peirce. He said that loss of the canine teeth, the teeth of the Chinese, or any rice-eating people, indicates a specialization,—that is, that we are being specialized in a certain direction. That is in opposition to a conception I had regarding the use of the teeth, which I would desire to speak about. It is not original with me, but the term "canine" or the dog-tooth, is a misnomer, as applied to the mammal called man.

If you watch the nut-eating animal called the gorilla, or the monkey, or any of that family, and put into his mouth a nut to crack, you will see that he takes it and carries it back of the bicuspids to the molars, and carefully places it there with his tongue and with the organs that surround it in the cheeks. He feels his way before he cracks the nut, and in feeling his way he passes the lower cuspid beneath the upper cuspid, and then cracks the nut. What does that teach us? The same thing that teaches us regarding man,—that the incisors are for incising or cutting; that the cuspids are not to tear, like a dog, for you do not tear your food, but you use them as a dowel. The gorilla, or the animal that has the longer cuspids, longer than man, places them so that when they pass each other they hold that nut in a position not to fracture the jaw, or as a dowel. He will not crack the nut until he has that cuspid in position with the other cuspid, and this was not indicated by the speaker last on the floor, whose remarks were so clever and interesting, and entirely truthful up to that point; but I think that point of observation is in error. Hence I say that mammals are not intended to tear their food, and those teeth should not be called canines, or dog-teeth. As the lower maxilla, the back part, the condyle of the inferior maxilla, slopes upward, the upper maxilla slopes to correspond with it, so you have a pressing of the two maxillæ in the act of mastication from the back part, and we have a pressing on the other part from the cuspidata.

*Dr. Barrett.*—If we take the Australian continent we find the dentition entirely modified. All of the animals, as far as I can learn, indigenous to the continent, were originally probably marsupial. The didelphia have a peculiar modification of the molar teeth in this, that the type is reversed,—that is, the typical number of molars is completely reversed. The carnivora indigenous to Australia are marsupial carnivora; the ungulata are marsupial ungulata. I do not believe that they all produce their young in the same way, but I think they have been modified to the type of marsupials. They reverse the order of the molars and the premolars; you find that constantly in the Australian continent, in those animals indigenous to the continent.

When we take that into consideration with some of the other facts in the paper, we find that it leads us to certain conclusions which cannot be avoided. Those conclusions you can draw for yourselves. It is for you to do it, and you can then understand and comprehend something of what the homologists are doing. The Australian continent is peculiar because the dentition is modified to the marsupial kind.

*Dr. James Truman.*—I would like to ask Professor Peirce one question. He said that there were thousands of human mouths where they have lost the wisdom tooth entirely. What evidence has he of that? He also stated that the canine tooth was the typical carnivorous tooth. I have always been taught that the sectorial tooth was the typical carnivorous tooth.

*Dr. Peirce.*—A remark in Dr. Thompson's paper was to the effect that Professors Cope and Allen stated that the typical carnivorous teeth were what we call the cuspid teeth and the sectorial molars. Those two were the typical teeth of the carnivora. I did state that they are the typical teeth of the carnivora; they are always present. The length or prominence of the cuspid tooth marks the carnivorous type. I stated there were thousands of families where the number of human teeth had been reduced to twenty-eight. I base my statement on this fact: I have at least twenty patients in my practice where that tooth never has and never will erupt. If I should have twenty individuals in my practice—very limited as it is—where that tooth never has and never will appear, I am justified in saying there are thousands of the human family where it never has and never will appear. So common is this occurrence that the scientific world recognizes it. It is noted in many scientific articles that the human family is fast losing four of its permanent teeth, largely the third molar, though in some families it is the lateral incisor. I do believe that the third molar is gradually disappearing from the human mouth, and if I may be allowed another moment, I would like to refer to the evidence of this. If we go back to the animals that are supposed to be ancestral to, or at least more closely allied to, the human family than others, we find first thirty-six teeth, then thirty-four, and then thirty-two. Now, we take the monkeys. Some of them have thirty-four teeth, and some of the lemurs thirty-six, and there is also an abundance of room between the last tooth and the ramus. We take the gorilla and the lower races of men, and we find thirty-two teeth all in place, with quite room for an additional tooth between the third molar and the ramus. Not only that, but we find the third molar equally large with the first and second. I have examined many jaws—some quite recently—for the benefit of Dr. Patrick. In many of them the third molar is fully as large as the first. In the human family (cultured) to-day the third molar is invariably smaller than either the first or the second. The conclusion naturally to be drawn is that there is a tendency to its suppression.

*Dr. James Truman.*—I would like to make another inquiry. You answer that the wisdom tooth, because it is not present in the jaw; you infer, therefore, that it is not in the jaw at all. Suppose you were to make an autopsy of that jaw and find the wisdom tooth in the ramus, what would you suppose?

*Dr. Peirce.*—There are hundreds of molars that have the germs started and are never completed. I find in my practice third molars coming through at fifty years of age, due to the absorption of superimposed tissue. They are imperfect in their development, which is an evidence of tendency to suppression. I think it is universally conceded that the third molar is invariably smaller than the first or second. The durability, too, is doubtful. Which tooth after the age of twenty-five is the one most frequently lost? The third molar. It is the perishable tooth subsequent to that age, or rather between the ages of twenty-five and forty.

*Dr. Barrett.*—Are you positive that any of the monkeys have a dentition of thirty-six teeth, and if you are, can you give me the class?

*Dr. Peirce.*—I alluded to the lemurs as having thirty-six. The monkeys, several varieties, have thirty-four.

*Dr. Truman.*—What means the excessive development of the cuspid teeth in the vegetable feeders, as in the camel?

*Dr. Peirce.*—The excessive development may be the transmission of an ancestral condition. Some of the vegetable feeders may not have been long enough such to suppress them. The camel, for instance, may have used his as prehensile organs. As compared with those of carnivorous animals, however, their characteristics are suppressed.

*Dr. Brophy.*—I understood the professor to say that the third molar in the human subject is invariably smaller than any of the others. Is that correct?

*Dr. Peirce.*—I said so,—yes.

*Dr. Brophy.*—Is it not a fact that the third molar is larger and broader, and the crown larger than the others?

*Dr. Peirce.*—Invariably it is smaller. Occasionally it is larger. Those are exceptional cases.

*Dr. Brophy.*—The discussion on this subject will not be lengthened by what I have to say; although I am not a close observer of the conditions that have been discussed, I have observed this: that the lateral incisor is almost as frequently found absent as the third molar; and in conversation with Dr. Black, not long ago, he agreed with me that the lateral incisor tooth was almost as often absent as the third molar. It may be that during this process the third molar will disappear in time. I am convinced, however, that the incisor has disappeared in certain families. It has been absent in three generations in some cases. What can we prove by that? It may not be in the exact line of scientific discussion that we are engaged in, but the question is, "How can we account for it?"

*Dr. Peirce.*—I want to make just one remark regarding the difference between the loss of the incisor and third molar. I simply give this as my own observation. Dr. Brophy said that there were families where the lateral incisor was absent. The deficiency of this tooth in either one or both parents is more likely, it seems to me, to be transmitted to the child than the deficiency of the third molar. The deficiency of the third molar, I deem, comes through an evolutionary process,—want of use and want of room,—and may or may not be deficient in children as ancestral tendencies are strong or weak.

*Dr. Morgan.*—He assumes that the loss of this incisor and of the molar is the result of the non-use of the tooth, if I understand his views,—that it is lost because there is no longer any use for it. What I want him to tell me is this: The inferior incisors are not lost. They are of less use than the upper, the superior incisors. They are of less importance for any of the purposes for which they are used, and yet we find them lost in the superior maxilla and they are retained in the inferior. If his theory be correct, they must be lost alike in both maxillæ. It seems to me that there should be a way to explain it.

*Dr. Peirce.*—The doctor is not correct when he says I assume the lateral incisor is lost for want of use. The third molar is subject to that law of specialization,—non-use,—but not the incisor.

*Dr. Morgan.*—Why not all of the upper teeth under the same law?

*Dr. Peirce.*—I can only give the results of the facts as they exist. I do not know why not. All through the animal world the loss of premolars is noticeable. Some of the monkeys have lost the premolars, not the third molars. Man has lost an additional premolar. The third molar is now in process of suppression for want of use and room.

*Dr. Rhein.*—The remarks made by Professor Morgan were very interesting to me, because they were just what was uppermost in my mind at the time,—that specialization of the human race, if it were tending to the loss of the teeth, could not very well involve the loss of any of the central incisors, or of any of the incisors, and the fact that we meet with the loss so frequently of the superior lateral incisors is undoubtedly a very interesting point in conjunction with this question. The argument put forth by Professor Morgan, that we do not find it in the inferior laterals, is a very strong one against the theory that its loss is in the same line as the other molars. I have had a theory in regard to the loss of the superior lateral incisors for a number of years, and I feel very strongly urged to present it here. The theory was suggested to me by a couple of cases where I could distinctly trace back, at some later day, a condition of double harelip in the family. We know that invariably where there is double harelip it involves right in that portion the germs of the lateral incisors, and it is not at all strange to find in such cases—and we have plenty of them—that the lateral incisors are absolutely lacking. We see cases in children where they have been operated on, and sometimes they have not even the central incisors. I have only in two cases out of a number I have observed been able to trace back any hereditary condition of this sort, but it seems a very natural thing that, if such a condition existed in the past, in the natural course of heredity it would be transmitted to the succeeding generations. I merely offer it as a suggestion towards the elucidation of this very interesting point in the discussion of this subject.

*Dr. Marshall.*—I would like to know if I understand Dr. Peirce to say that man had lost a premolar?

*Dr. Peirce.*—As compared with the dentition of the monkey.

*Dr. Marshall.*—Does he mean that we are really descended from the monkey?

*Dr. Peirce.*—There is not time to discuss that now.

*Dr. Marshall.*—I should like, for the edification of this body, that he should give us some facts about this. I have not yet learned that there were three premolars in any prehistoric skull. I never heard such a claim made before.

Subject passed.

*Dr. Barrett.*—I congratulate the association upon the fact of its sitting and listening to such a technical discussion for so long.

### DISEASES OF THE ORAL MUCOUS MEMBRANE.

BY DR. J. D. PATTERSON.

This subject has been very much neglected, because its importance has been underrated. Until the agitation brought about by investigation of the disease called pyorrhœa alveolaris there had been little attention paid to diseases of the mucous membrane. Since then investigators have recognized more the importance to the dental practitioner of a thorough knowledge of the etiology and pathology of these complaints.

The inception of the irritation by which we have diseases of the oral mucous membrane may be divided as follows: Accidental, constitutional, and hereditary.

I desire in this article to specially call your attention to some of the accidental causes. They are very numerous. The irritation may be mechanical alone, or it may be from the invasion of microorganisms, from absence of hygienic care, from disease, from infection, or from functional disturbance without mechanical aid.

We will first consider the cause of disease by an arrest of its function. The dental profession has not given thought to this line of investigation so much as to the search for constitutional conditions, and therefore, I believe, have signally failed to explain the etiology of certain symptoms, especially in the early stages of pyorrhœa.

In glancing at the anatomy of the membrane, it may be described as follows: A layer of epithelium; a connective tissue framework and glands underneath, separating the epithelial layer and provided with blood-vessels, lymphatics, and nerves. Whatever interferes with and changes the normal condition of the membrane may overcome its safeguards, and disease is the result.

Any marked deviation from the normal heat of the tissues —namely, 98°—results in morbid changes. Heat-production goes on all over the body. When any of the tissues are subject to cold, the production is stopped, and morbid action may occur. In the mouth the contraction of the membrane is the first step, accompanied by stoppage of the mucous secretion. We have increased nutrition; an over-production of the mucous secretion, a slightly reddened tissue from the same cause, an abundant diffusion of serum escapes. As the inflammation progresses, we see the discharge becoming a purulent one. Micro-organisms now make their invasion, and assist in the advancing stages of the disease. These soon extend below the epithelium and the membrane swells and thickens.

Let it be borne in mind that up to an advanced stage there is not, of necessity, any presence of pathogenic or non-pathogenic micro-organisms.

The hereditary condition of tissue and constitutional environments are in these cases merely predisposing factors. In some cases it is not to be doubted that constitutional conditions figure largely, and the mucous membrane is at the inception of the disease involved; but there are greater factors which explain other symptoms without seeking in the field of bodily conditions.

I pass now to another irritant,—the prevalent practice of mouth-breathing. Leaving out of the question the change in the temperature, we find that the air inspired rapidly dries the surface of the mucous membrane, stopping the mucus-ducts until the same train of symptoms from destroyed functions already described is established. When a subject is presented who from one cause or another breathes through the mouth, the operator may at once predict the condition of the mucous membrane. Here we have again a numerous class of cases where undeniably the disease has arisen from the gradual drying of the naturally moist lubricating surface by the introduction of an atmosphere which is not normal to the tissue.

The condition that is described as arising from the interference with the functions is not a result of mouth-breathing alone. It may result from different causes. It is seen in infection from catarrhal, croupous, diphtheritic, or tubercular surroundings. They are superinduced sometimes by medicinal treatment, by mechanical irritants, and by uncleanliness. We find that while the cause of the described conditions may be varied, and that the cause will affect the malignancy of the disease, yet the symptoms are very similar.

This condition is a disturbed function. One of the chief symptoms is an abnormal secretion of the mucous pus.

In the oral cavity the train of symptoms we have described meets with conditions which rapidly further the inflammatory process. We refer to the dental organs. These symptoms continue, and finally develop into what is called pyorrhœa alveolaris, which is a condition which naturally results from this.

In diseases of the membrane of the mouth, resulting in what is familiarly known as pyorrhœa alveolaris, different authors are opposed to the term "catarrhal" in describing the clinical appearance. They consider catarrh a special disease of certain membranes, which ultimately leads to ulceration. It is not so at all. It is simply a condition of discharge, and not a disease of special cause.

Another somewhat prevalent idea entertained by the profession is that there is a peculiar systemic condition under which the patient becomes liable to catarrhal inflammation.

Dr. Bosworth says, "I know of no such condition, and there is no good ground for the assertion. My clinical observation also fails to justify this view in any manner." ("Diseases of the Throat and Nose," p. 101.)

Another argument against classifying these diseases of the oral mucous membrane as "catarrhal" comes from those who lay great stress upon climatic influence. They find in climates where catarrh is almost unknown, many cases of pyorrhœa, and, on the contrary, where catarrhs are endemic, find no increase in the number of those suffering from discharge from the oral mucous membrane.

I think this statement should be considered a very loose one. It was formerly supposed that more of these diseases were in America than in Europe, but this has been disproved. There can be no question that the change of climate works wonders in catarrhal trouble, but that climatic influence is an important factor in its causation is open to question.

In leaving the question for your discussion, I leave with you the following considerations:

*First.* That the inception of disease in the membrane, where the cause is obscure, is often to be found in the arrest of function in that membrane by shock, by catching cold, or by mouth-breathing, by which the moist lubricated membrane is destroyed, rather than by hereditary conditions, or the invasion of micro-organisms.

*Second.* That the irritated mucous membrane, which we find in abnormal quantities, is not pathognomonic of any special irritation, but is simply a condition.

*Third.* That this condition is properly termed catarrh.

*Fourth.* Catarrh is not a special disease; merely a condition of disease.

*Fifth.* That the name pyorrhœa alveolaris simply denotes a stage of this catarrh in the oral mucous membrane, and may result from a great variety of causes.

### PHAGOCYTOSIS.

BY DR. H. A. SMITH.

A correct knowledge of the life of cells forms the basis of physiology. A cell may be defined as a nucleated mass of protoplasm of microscopic size, which possesses sufficient individuality to form a life history of its own; originating from some pre-existing cell, it grows, reproduces itself, and dies.

The file of physiological activities of protoplasm may be studied in the amœba. They are,—

1. Power of spontaneous movement, called amœboid movement.
2. Irritability and power of response to stimuli.
3. Nutritive powers,—the ability to take in food and build up tissues by assimilation and rejecting what is not assimilated.
4. Retro-active powers.
5. Decay and death of cells.

But our purpose is to call attention to the white corpuscles or leucocytes of the human blood, and refer especially to their amœboid movement, first described by Wharton Jones. These leucocytes are simple sperical masses of protoplasm, without cell wall, "but in spite of their simple organization, they act as other creatures do."

Their power of spontaneous movement is accomplished by the mass lengthening in one direction, and then the whole mass flowing into the process, and thus the cell changes position.

Its nutritive power is manifested through this prolongation or tentacle seizing the food and drawing it into its mass. Because of those vital or physiological qualities, the white blood-cells have been called by the eminent Russian zoologist, Machinkoff, "phagocytes," or devourers. In the published scientific literature of the day concerning those cells, they have been called eating cells, wandering cells, chimney-sweeps of the blood, etc.,—names indicating that, in a way peculiar to themselves, they assist in purifying the blood, having the ability to seize upon the waste products of the system.

It is also supposed the phagocytes prey upon the small foreign bodies in the blood, organic or inorganic, dead or living, including diseased germs and all invading bacteria.

The plausibility of Mechinkoff's phagocytic theory and its acceptance by some of the known morphologists of the world has suggested that perhaps some of the mysterious phenomena observed in the treatment of disease by the dental practitioner might be explained by the drawing function of the leucocytes.

In the January number of the Buffalo *Dental Journal*, Dr. Frank W. Low has given us an interesting paper entitled "Phagocytosis." Following an explanation of the theory he says, "In the existence of the phagocytes we have an intelligent explanation of the successful outcome of much of the careless root-work which is undoubtedly being done the whole country over."

He believes that teeth, if treated with dry, hot air and mechanical cleansing, as practised by some operators, speedily assume healthy relations with the surrounding tissues.

The phagocyte cells act as factors in devouring all bacteria, both in the pulp-canal and in the tissues about the apex of the roots. Dr. Low ventures the opinion that phagocytes may penetrate the dental tubuli of the root from the pulp-canal It is doubtful if the smallest blood corpuscle, average size $\frac{1}{2500}$ inch, could enter those tubuli, especially if the root-canal is surrounded with a dense layer of dentine, as recently described by Professor Miller.

If the dental pulp is invaded by putrefactive micro-organisms, through a point of exposure, or by way of the vessels entering the pulp through the foramen, those wandering cells may and probably do enter with the blood supplied to the pulp.

Dr. Low believes that when stasis is complete, the battle goes against the phagocytes, because reinforcements cannot come to the rescue, while the bacteria proliferate without hinderance in the dead pulp-tissue.

Pulp sometimes dies from injury in teeth, the crowns of which are sound. In those cases the pulp may or may not become putrefactive. In those cases in which the pulp atrophies or remains moist and, as we say, sweet, it may be assumed that the bacteria circulating in the blood and brought to the injured tissues at the apical space have not entered the pulp.

In cases in which the injury has been severe, and inflammation confined more nearly about the apex of the root, we are less likely to have a putrefactive pulp and dentinal abscess. The severe injury is followed by prompt and decided inflammatory reaction, in which the leucocytes pass out freely into the inflamed area and readily devour the pyogenic cocci always present in the damaged tissue.

Dr. Low gives the details of several bacteriological experiments with teeth containing putrefactive pulps, and concludes: "But one inference can be drawn from the results observed,—that the phagocytic cells act as the factors of alimentation in devouring all bacteria in the pulp-canal and the tissues about the apex of the root."

The cause of the disease known as pyorrhœa alveolaris has not yet been determined. It is, however, generally agreed that pathogenic bacteria are always present in the disease. As a means of cure, very much has been claimed for the heroic surgical treatment, as practised by the late Dr. Riggs, and others since his day. How the injury to the tissues resulting from this mode of treatment exercises a curative effect has been an enigma. May it not be naturally explained by the phagocytic action of the leucocytes which crowd in when the fibrinous degree of inflammation following tissual injury is reached? The bacteria always present, both specific and non-specific, are devoured, and naturally the inflammatory process ends with the formation of new or cicatricial tissue. Then it is said you have a cure.

If pyorrhœa is caused by a micro-organism, and is an infectious disease, we have here an instance where the inflammation, excited by a particular microbe, becomes, through the medium of leucocytes, the cause of its own destruction.

We have been taught to place great reliance upon the so-called vital-resisting powers of the tissues. No doubt the living tissues exert a great influence in checking the development of bacteria in the diseased conditions I have mentioned, as well as in many others we are called upon to treat; but it is only when we fully recognize the phagocytic action of the white blood corpuscles that much which is obscure in treatment is rendered comprehensible.

However, a belief in phagocytosis should not lead us to relax in the least the application of the most thorough antiseptic methods in our daily practice. The study of antisepsis and the intelligent use of antiseptic agents has done more than all else to cause dentistry to be classed as an art.

Antisepsis and phagocytosis, then, are both important factors in the treatment and cure of all germ-diseases, and should both alike receive our careful attention.

DISCUSSION.

*Dr. Rhein.*—The paper as read by Dr. Patterson, in going over the forms of disease of the mucous membrane and describing the various methods by which diseases of this membrane are brought about, was very interesting, and I, for my part, have no doubt that such cases are frequently met with; but in all they are bound to yield very readily to treatment. They are simply localized disturbances, and ought not to bring much difficulty to the practitioner, if they are properly diagnosticated. But here is where I draw the line. I think, and have always thought, that Dr. Patterson's view in regard to the disease that is called pyorrhœa alveolaris is that he does not take the proper view of the important part constitutional disturbances play in the cases of this kind that come under our notice.

After a careful hearing of his paper, the effect that was left upon me was that it was a very simple matter, and that all of these forms ought to yield readily to treatment. They do not do so, and it is almost imperative that we find some other factor as their cause. I differ with him on one point, and that is, that in the inception of this trouble we find an increased activity in the parts.

In the observations I have made, I found exactly the opposite condition. I have found that there is a decided stasis in the blood-supply, and that is where I believe we must look to find the real secret of the origin. If he is correct in his assertion that there is an increased activity (there may be in some of the forms), the rest of his assertions could possibly follow; but if he is wrong at the outset, and there is no increased activity, but, on the contrary, there is a diminished activity, why the whole fabric of his argument falls to the ground.

With the remarks which he made that we generally find these forms of disease in every climate and under all conditions, I agree, and it bears out the line of reasoning that I would like to pursue, —that it is due entirely to a perverted function of the general system, manifesting itself locally in these parts.

He dwells particularly on that point, and it supports the fact which I would bring up in contradistinction to his arguments. It is very difficult to argue on this matter without taking in both of these papers.

The last paper was very admirable, as far as it bore on this subject, and I think tends to sustain the line of argument that I am following.

One of the points that Dr. Smith offered was in regard to the results that are so often claimed for the violent treatment as originally demonstrated by Dr. Riggs. I think we all know from experience, and from what we have seen, that there is no question that very beneficial results have followed from this course of treatment. This was mechanical, extending to the roots, and the theory to be advanced as to the reason why this may be beneficial is the fact that it goes deep enough to strike all the tissue that is in that condition of inactivity, and the stimulation which follows from this violent action brings about increased activity to the parts that we can see in the operation of implantation, when it is performed.

It is following the same line of increased activity, as long as you produce sufficient irritation. I believe that the time ought to be near at hand when we should get a better name for this type of trouble that we meet so frequently. There are so many varied forms of purulent discharges that arise from dissimilar causes that it is almost impossible to discuss this subject without leaving a place open for some one to really give actual cases in practice that are exactly different from any line of reasoning that a speaker cares to take in the discussion of this subject, showing the fact that any disturbance of the functional activity can produce this trouble.

There are a great many cases of nothing more than alveolar abscesses that are often confounded with pyorrhœa alveolaris, and are treated for that without ever looking to the real source of the trouble. I believe that a large majority of cases are constantly treated for pyorrhœa where the pulp of the tooth is simply dead and this death of the pulp has not been properly diagnosticated.

I offer this suggestion because I have found in a very large percentage of cases that no one would suspect the pulp of not being in a good, healthy condition,—where there was no external abrasion of the teeth, where the teeth were to all appearances sound, where they did not show any opacity,—yet, on a more careful examination of it, the pulp in those teeth would be found to be dead. In some, irritation resulting from the death of the pulp was sufficient to cause the conditions we find in pyorrhœa alveolaris.

Granting such a condition of affairs to exist, we all know that radical treatment necessitates the removing of the *débris* in the tooth. I throw out this suggestion for the members, because I believe very few men in practice have an idea of the number of lesions of this character that exist, and the question is almost entirely ignored in making a diagnosis, because of the normal appearance of the teeth.

*Dr. Barrett.*—It is not every day that we can have so many papers of such great interest and value presented to the Association, and have them listened to with so much patience as we have witnessed this morning.

The first paper that is now under discussion is of very great interest, but I have nothing special to say upon that, other than to congratulate the Association upon having the opportunity to listen to it.

It has long been a problem in human physiology as to the leucocytes of the blood. What their peculiar office is has not been determined. What has been their mission is not known, but physiologists know that they were not created in vain. It is extremely probable that the theory which has been advanced and was urged last summer in Berlin is true. Professor Lister there read a paper before the International Medical Congress which is of great interest. In it he took occasion to recede from the ground he took nine years before, and to declare that in his opinion an entire aseptic condition could not be secured as long as there was the existence of bacteria in the blood; but we know that one who is in ordinary health is generally in immunity from those diseases, that the system seems to be proof against this infection. How can it be?

In other conditions we find that this is not the case. There must be something or other that shall act to protect the system. We know that as we stand and sit here that microbes, pathogenic and non-pathogenic, are in the air. How is it that we are not inoculated with them? How is it that the bacteria do not find lodgement in the human system? Why does it not produce these effects? There must be some means of protecting the system.

It is extremely probable, although I do not believe that it is established as a scientific fact, yet it is probable that this exists.

The leucocytes are in a mass of jelly-like consistency that seems about to melt. It has been observed that certain organisms have been seen within the body of an amœboid cell, especially in the rod-like bacteria. It envelops itself about the bacteria, and you find the rod projecting at both ends,—the rod-like bacteria projecting at each end from the body of the amœboid cell, while digestion was going on within the body. As this becomes digested the ends have dropped off and been lost, while the central portion was absolutely absorbed.

An amœboid cell may digest organic matter of this kind simply by enveloping it. That is the method when the blood is in a proper condition, that they will envelop it and digest it, and so destroy and limit the spread of the bacteria.

Here is an experiment that was performed recently. You know the frog is not subject to inoculation for anthrax; but anthrax spores have been enveloped within the pith of a piece of elder, and introduced between the epidermis of the frog. What does that mean? It means that the leucocytes cannot penetrate the pith, but that the serum of the blood can do so. What was the result? The leucocytes being excluded, the disease continued; but the moment the spores reached the surface and the leucocytes could obtain access, a change occurred, and they were absorbed and destroyed. The experiment seems to be almost conclusive.

*Dr. Cravens.*—It is a pleasure to me to discover that the gentlemen are struggling to explain this subject. It is a pleasure to know that in doing so they have been in a measure successful.

If success has attended immediate root-filling, it certainly requires no ability. I presume that all practitioners would gladly welcome the phagocytes as elements of success. Some gentlemen first denied that immediate root-filling was possible. Now they have discovered why it has been successful, and they immediately take away from those who advocated and practised that method all the credit of it, and say that in spite of our bad manipulation, through the assistance of the phagocytes, the operation of immediate root-filling has been successful.

*Dr. Waters*, of Boston.—The author of the first paper agreed with me in his experience that all these lesions arising from micro-organisms were preceded by a disturbed condition of the mucous membrane, and that state he ascribed to certain other disturbances.

When a child is born, the surface of the body is pink. It requires a change of conditions and also of the atmosphere to alter the color of the skin and make it pale, and in proportion to that paleness is the health of the child. So long as the skin remains pink and warm the child continues to be healthy. When the skin bleaches, then catarrhal symptoms are present with liability to the development of micro-organisms. If the child lives so as to be able to go to school, a glance of the master's eye will sometimes increase the circulation.

Atmospheric as well as mental conditions have their influence too. I find our east winds, charged with the moisture of the ocean, have a depressing influence upon the functions of the skin, to cause the contraction and disturbance in its action. When we have these antagonisms the pores of the body are closed. What becomes of the carbonic acid that is generated? It is contained in the body, but not in the form of carbonic acid. It unites with the other acids of the blood; it is associated with albumen. When carbonic acid is united with soda it passes from the body through the kidneys. When you get below a proper amount of alkalinity in the blood you have then the condition which the essayist speaks of in his paper, and not until then. It may be local. No case of consumption ever existed that did not have for its inception a disturbance of the functions of the skin, or possibly, I might say, some external influence which reacted upon the skin and produced derangement.

*Dr. Leroy.*—I would like to ask some gentleman in the room whether pyorrhœa alveolaris affects devitalized teeth? My attention has been directed to the same.

*Dr. Rhein.*—I would say that the worst case of so-called pyorrhœa of the teeth, where there was a permanent discharge, that I ever saw was where the pulps were devitalized.

*Dr. Morgan.*—Whenever the periosteal tissue is destroyed, you have the destruction of the tooth, which produces alveolar abscesses.

*Dr. Crawford.*—I doubt very seriously whether a genuine case of pyorrhœa alveolaris has ever developed upon a tooth or the tissues in which a tooth is located after the death of the nerve. I have made some observations on that line, but have not reduced them to proper order to be presented. I have had some cases cited to me in opposition to that theory, but I have invariably found this condition to exist.

*Dr. Patterson.*—I would like to state, in answer to the gentleman who spoke first, that I have never at any time doubted but that constitutional environments must be taken into account in considering diseases of the mucous membrane. I did not give much attention to that in my paper, because that was not my point. This was to direct attention to what was the incipient cause in obscure irritations of mucous membranes which resulted in diseases. I do not doubt that a large number of the lesions of the mucous membrane can be ascribed to constitutional causes. Dr. Rhein was in error in one remark he made. He stated that I said that there was increased nutrition after function was destroyed. So there is. It is the same in all cases of inflammation. Then comes the stasis which he referred to, and the loss of nutrition, but after irritation there is loss of function, then a stoppage of circulation, after which we see increased diffusion and discharges, and all the stages of inflammation. That is all I have to say on the question.

*Dr. Smith.*—I have already occupied too much time for the section, and as chairman I wish to thank the association for their attention to its protracted discussion.

Report of Section VII., "Anatomy, Pathology, and Surgery, was presented by Dr. T. W. Brophy, chairman.

The following paper was read by Dr. Barrett, of Buffalo:

### A PLEA FOR CONSERVATISM.

BY DR. W. C. BARRETT, BUFFALO.

It seems to me time that we began to consider the subject of antiseptic dental treatment from a more rational stand-point. Microbes cannot be utterly excluded from the mouth. They will penetrate cavities of decay despite the greatest precautions. Utter asepticism in any portion of the human system is a dream that has no foundation in fact. At the International Medical Congress in London, in 1881, Sir Joseph Lister, who has given his name to a method of operative procedure, announced that the horrors of surgical interference were abolished, and that by means of the carbolic spray it was possible to operate under entirely aseptic conditions. But Ziegler and Bantock and Tait scored even greater successes than did Lister. They spurned his spray, being only sedulously careful as to cleanliness and irrigation.

At the Congress held in Berlin last summer, we who had listened to Professor Lister with so much suppressed emotion only nine years before, were astounded to hear him now say, "As regards the spray, I feel ashamed that I should ever have recommended it for the purpose of destroying the microbes of the air."

The practice recommended by some of our most intelligent dentists in the treatment of root-canals, it seems to me, looks towards the entire exclusion of microbes. Everything is managed with this end in view. The utmost stress is laid upon the most minute parts, while other needed precautions are neglected. In cases of alveolar abscesses, we hear of nothing save detergents and antiseptics. It seems to be taken for granted that the microbe can be exterminated. Nothing else need be done. In the capping of exposed pulps, asepticism is made the only condition of success.

I wish again to say that I am not urging indifference to antiseptic treatment. I believe it to be absolutely essential to successful practice; but I do not think that it is the sole factor and the only thing to be looked after. I do not believe that the entire absence of all organism can ever be assured. I do not hold it possible to secure a complete aseptic condition by any external means or agent. We cannot control the processes of nature. We can only influence them to a certain degree. We cannot, for instance, furnish the proximate principles of the body from without. We can only supply the proper pabulum, and from that allow the nutritive system to select and prepare its own material. The attempt to harden the bones of the body by giving phosphate of lime is a hopeless task, and it is quite as much so to undertake the extermination from the tissues and organs of the body of all the micro-organisms, either of pathogenic or non-pathogenic nature.

The oral cavity is never without microbes of various kinds. The multiplication of remedy and the duplication and reduplication of methods will not compass the impossible. The more complicated the processes employed, the greater the opportunity for error. The entire sterilization of dental instruments is impracticable. We may approximate it, but it is hopeless to attempt to destroy all spores without ruining the instrument. The germs of different fungi are too numerous and too widely diffused to be entirely destroyed. Wherever air enters, there goes the possibility of infection, and air cannot be utterly excluded. Within the tissues, diffused throughout the blood, penetrating everywhere, the spores of the fungi are found in health as well as in disease. There is no immunity from infection when an organ or tissue shall ever have taken on a condition of atony. The microbe has all lands for his own, and it is useless to attempt to fly from his presence.

Does this absolve us from all attempt at asepticism? By no means. The very fact of the universality of the microbe should stimulate us to greater precaution. That instruments may not be absolutely sterile is no excuse for the almost universal and criminal neglect of proper precaution.

How many operators keep a sterilizing apparatus and faithfully use it? It is of no material use merely to dip a point into some solution and then call it sterilized. How many have a special apparatus for the purpose? And yet no man should be permitted to practise without something of the kind.

While we cannot utterly banish micro-organisms, we have no right to be inoculating with specific pathogenic bacteria. I have seen many cases in which a previously healthy mouth became diseased after the filling of a tooth by the carelessness of some ignorant dentist.

In a condition of entire health, nature has made provision for the entire destruction of invading bacteria. Whether the theory of Metchinkoff be true, and the peculiar power to digest and devour them rests in the leucocytes of the blood, or whether microbicidic action can be found in some other function, we cannot positively state; but we do know that until the tone of the system be in some way depressed, there is immunity from the usual pathogenic organism.

That the spores of different species vary in virulence is, of course, a fact; but it does not affect the truth of the principle. A tooth that has lost its pulp cannot be said to be in a normal condition; yet in the healthy mouth, if proper precautions have been taken, and if the right mechanical manner for its protection shall have been employed, we know that it is secure against the attack of disease under ordinary circumstances. We know that in many instances a pulp has been destroyed in the most slovenly way imaginable, without any regard even for decent cleanliness, and fillings have been inserted over devitalized pulps without even an attempt to remove the contents of the root-canal, and it has remained in apparent good condition for years.

We know that until within a comparatively short time the roots of teeth were filled without antiseptic precautions, because the condition was not then comprehended, and yet they were sometimes well saved. Westcott and Dwinelle, and their compeers, filled nerve-canals before microbes and antiseptics were dreamed of, and they did it with success too. Antiseptic treatment is not, then, always an essential; but Westcott and Dwinelle were not able to save as large a number of cases, nor were they, with their incomplete comprehension of pathology which belonged to the fifth and sixth decades of the century, warranted in attempting the salvation of a class of teeth which, in the light of the further knowledge of this tenth decade, we do not hesitate to fill.

I care little for coagulants or non-coagulants. Coagulation is certain in albumen when exposed to external agencies, and all the non-coagulants in the world cannot prevent it, except by its practical destruction. I care not to exclude all organisms, for I know that to be impossible. There are many which are not pathogenic in their character. It is idle to talk of danger from amœbaic, deep-seated organisms, for they are usually harmless, and if they were not, they could not be avoided. I care not that every instrument that enters the pulp-chamber shall have been heated to a red heat, which is the only way completely to secure sterilization. We know that the material with which the root is filled is never made sterile, and if the manipulation is what it should be there is no necessity for it. We know that utter asepticism of the mouth is a hopeless dream, even if it were desirable; but we also know that cleanliness and dryness are essential, and we know that these are within the reach of every careful operator. We know, too, that comparative freedom from pathogenic microbes is readily attainable, and the dentist who does not secure this is unworthy the name. We know that a state of asepticism is not the only one upon which the health of the tissues depends, and it is quite as excusable to neglect one necessary precaution as another.

So I again protest against the tone of some of our modern teachings that seems to ignore all that is not new and unheard-of by the average dentist, and that affects the obscure and the occult. I am willing to sacrifice my reputation for scholarship to that of good sense. I would rather have the approval of those who are really intelligent than the applause of any number of groundlings.

If I can be known as a safe, reliable, conservative, level-headed practitioner, I will resign all claims to daring, experimental, brilliant empiricism. I recognize the fact that it is necessary that there should be innovations and innovators, but I do not desire to be the patient of one. I honor those who by patient, laborious investigations elucidate the incontestable truths of science, but I cannot give my approbation to him who catches up the half of an idea, and by an absurd extravagance manages to taint even the best cause. Therefore, while I eagerly seize upon new truths, I will try to prove all things, and especially to hold fast to that which is good.

Adjourned.

